# Integrated management of mandibular fractures and traumatic dental injuries: a literature review and clinical algorithm proposal

**DOI:** 10.3389/froh.2026.1887405

**Published:** 2026-08-03

**Authors:** Edoardo Staderini, Raffaella Castagnola, Irene Cavalcanti, Marianna Balacco, Anastasio Spera, Cosimo Rupe, Gioele Gioco, Massimo Cordaro

**Affiliations:** 1 School of Dentistry, Catholic University of the Sacred Heart, Rome, Italy; 2 Department of Orthodontics, IRCCS “A. Gemelli” University Polyclinic Foundation, Rome, Italy; 3 Institute of Dental Clinic, A. Gemelli University Polyclinic IRCCS, Catholic University of the Sacred Heart, Rome, Italy

**Keywords:** clinical algorithm, dental trauma, intermaxillary fixation, mandibular fracture, maxillofacial trauma, open reduction and internal fixation, traumatic dental injuries

## Abstract

**Background:**

Mandibular fractures are among the most common maxillofacial injuries and are frequently associated with traumatic dental injuries (TDIs), creating combined skeletal, occlusal, periodontal, and pulpal treatment priorities. Integrated protocols addressing both the fracture and the dental component remain limited.

**Methods:**

The present literature review was conducted by means of a structured search of PubMed/MEDLINE, Scopus, and Web of Science Core Collection from 2000 to 2026, complemented by manual reference-list screening. A structured Population, Intervention/Exposure, Comparison, and Outcomes (PICO) framework and predefined eligibility criteria were added, and an overall assessment of methodological quality was undertaken for the included primary studies to improve transparency.

**Results:**

Mandibular fractures range from minimally displaced injuries with preserved occlusion to complex patterns associated with malocclusion, neurosensory impairment, temporomandibular joint dysfunction, and concomitant TDIs. The evidence supports individualized decision-making based on fracture displacement, occlusal stability, patient age, dentition status, tooth prognosis, and biological priorities of periodontal and pulpal healing. Teeth in the fracture line should not be extracted routinely; retention is generally favored when the tooth is intact, strategically useful for occlusion, and not infected, whereas extraction is indicated in cases of vertical/root fracture, advanced periodontal disease, severe caries, infection, or interference with reduction/fixation.

**Conclusion:**

Combined dento-mandibular trauma requires coordinated interdisciplinary management. A revised algorithm is proposed to integrate AO-CMF fracture principles with International Association of Dental Traumatology (IADT)-based dental trauma care, emphasizing systematic dental screening, selective imaging, safe fixation planning, biologically appropriate splinting, staged endodontic decision-making, and long-term follow-up for pulp necrosis, resorption, ankylosis, malocclusion, and neurosensory complications.

## Introduction

Mandibular fractures represent one of the most common forms of maxillofacial trauma (1–3). Mandibular fractures account for approximately 70% of facial fractures (2). The structural strength of the symphysis and the relative fragility of the condyle both act as a defense mechanism against traumatic injury, protecting mandibular bone integrity (4). Additionally, normal occlusion and an intact dentoalveolar system with effective muscular support reduce fracture risk, whereas pathological conditions such as osteolytic lesions or impacted teeth increase mandibular vulnerability to trauma (4, 5).

According to Zhou et al., dental injuries were identified in 43.5% of patients with a single mandibular fracture, involving 509 teeth and an average of approximately four injured teeth per affected patient (6). In pediatric populations, Kannari et al. reported dental injuries in 34.7% of patients with mandibular fractures, with loss of at least one tooth in approximately 10% of cases, often occurring at the time of the traumatic event (7). The pattern of associated injury also depends on whether a mandibular fracture is direct or indirect: direct fractures occur at the site of impact, whereas indirect fractures typically involve the condyle and result from trauma to the contralateral parasymphyseal region (4). Indeed, mandibular injuries are rarely encountered as isolated events and often present as complex clinical scenarios associated with tooth fractures and/or luxations (8, 9). In such conditions, the accuracy of the initial clinical assessment and the appropriateness of early therapeutic decisions play a more decisive role in prognosis than the promptness of invasive treatment alone (9).

In daily clinical practice, mandibular fractures show a wide spectrum of severity, ranging from minimally displaced fractures with preserved occlusion to complex injuries associated with functional impairment, neurosensory deficits, and temporomandibular joint (TMJ) involvement (1, 10). During initial triage, key red flags include airway compromise, uncontrolled bleeding, severe malocclusion, inability to tolerate oral intake, TDIs, suspected polytrauma, and progressive swelling or infection; these factors inform the urgency of referral and the treatment setting (6, 11). In the event of a TDI, factors such as tooth position and mobility, tenderness to percussion or contact, sulcular bleeding, soft-tissue findings, and occlusion should also be assessed (12).

According to AO-CMF (Arbeitsgemeinschaft für Osteosynthesefragen) principles, mandibular fractures may be classified as favorable or unfavorable based on the relationship between the fracture line orientation and the direction of masticatory muscle forces. In favorable fractures, muscular pull contributes to inter-fragmentary stability and approximation, whereas unfavorable fractures exhibit a fracture line that promotes displacement of the fragments under muscular action (13). The AO-CMF classification also categorizes mandibular fractures into condylar- and non-condylar fractures (13).

Early management of mandibular trauma is directed toward preserving the integrity of the masticatory system and temporomandibular joint function and, in growing patients, preventing growth disturbances (10, 14). For mandibular fractures associated with TDIs, management should be coordinated to prevent treatment of one condition from adversely affecting the other (6).

Despite their frequency, evidence-based treatment algorithms remain limited. Clinical management is largely guided by fracture pattern, patient age, occlusal stability, and functional impairment, with complication profiles described across different settings and populations (1, 15, 16).

In clinical practice, the initial question is not only how the fracture should be stabilized, but also whether the fracture pattern and the patient's condition—including occlusion, airway status, pain control, and associated dental injuries—require urgent referral or immediate stabilization (11).

## Aim of the review

The aim of this literature review is to synthesize the available evidence on the combined management of mandibular fractures and traumatic dental injuries (TDIs), with particular emphasis on the clinical interaction between fracture stabilization, teeth within the fracture line, dental splinting, endodontic timing, and long-term dental prognosis.

A secondary objective is to propose a clinically oriented, integrated decision-making algorithm that links AO-CMF fracture principles with IADT-based dental trauma management, rather than presenting mandibular fractures and TDIs as parallel but separate entities.

Each traumatic injury is addressed following a consistent structure that mirrors real-life clinical reasoning, explicitly addressing when radiographic examination is required, what must be evaluated clinically, which therapeutic actions are indicated, and what parameters should be monitored over time. This approach aims to translate evidence-based knowledge into a schematic and clinically oriented algorithm (11, 12, 17).

## Materials and methods

The present study is a literature review and clinical algorithm proposal. However, to improve transparency and reproducibility, the methodological approach was strengthened through a structured PICO framework, predefined eligibility criteria, and an overall assessment of methodological quality for the included primary studies.

### PICO framework

**Population:** patients with mandibular fractures, with or without associated traumatic dental injuries (TDIs). **Intervention/exposure:** conservative or functional treatment, intermaxillary/maxillomandibular fixation (IMF/MMF), open reduction and internal fixation (ORIF), dental splinting, tooth retention or extraction in the fracture line, and endodontic monitoring/treatment. **Comparison:** surgical vs. non-surgical fracture management, immediate vs. delayed dental management, and tooth retention vs. extraction when applicable. **Outcomes:** occlusal stability, fracture healing, infection, neurosensory function, temporomandibular joint function, pulp survival, root resorption, ankylosis, tooth survival, and need for delayed extraction or endodontic treatment.

### Search strategy

A structured literature search was conducted in PubMed/MEDLINE, Scopus, and Web of Science Core Collection. The search covered publications from 2000 to 2026. Seminal pre-2000 studies identified through manual reference-list screening were retained when providing clinically relevant information not adequately represented in more recent publications. Three complementary PubMed/MEDLINE search blocks were used: (1) combined mandibular fracture and traumatic dental injury terms, (2) teeth in the fracture line and tooth retention/extraction terms, and (3) ORIF, closed/conservative treatment, IMF/MMF, and outcomes terms. Equivalent main and teeth-in-fracture-line searches were performed in Scopus and Web of Science. Search terms included combinations of: “mandibular fracture”, “mandible fracture”, “mandibular trauma”, “condylar fracture”, “mandibular angle fracture”, “mandibular body fracture”, “symphysis fracture”, “parasymphysis fracture”, “ramus fracture”, “coronoid process fracture”, “traumatic dental injury”, “dental trauma”, “dentoalveolar injury”, “dentoalveolar fracture”, “tooth in fracture line”, “teeth in the fracture line”, “dental splinting”, “avulsion”, “luxation”, “pulp necrosis”, “root resorption”, “intermaxillary fixation”, “maxillomandibular fixation”, and “open reduction and internal fixation”. Database searches yielded 2,508 records (PubMed/MEDLINE *n* = 1,340; Scopus *n* = 832; Web of Science *n* = 336). Seven additional sources were identified through manual reference-list screening and targeted guideline/clinical-resource searches. After duplicate removal, 2,083 records/sources were screened. The detailed search strings and database-specific counts are reported in Supplementary Table S3.

### Eligibility criteria

Eligible records included systematic reviews, meta-analyses, randomized clinical trials, prospective and retrospective clinical studies, cohort studies, case series with clinically relevant management information, epidemiological studies, and international guidelines addressing mandibular fractures, TDIs, teeth in fracture lines, fracture fixation, dental splinting, endodontic timing, or follow-up. Exclusion criteria were animal or *in-vitro* studies without direct clinical relevance, editorials or letters without original or guideline-level content, articles not related to mandibular fractures or TDIs, and studies in which the cited statement was not supported by the reported results.

### Assessment of relevance, validity and data extraction

Because of the heterogeneity of fracture patterns, dental injuries, age groups, interventions, and outcomes, a quantitative meta-analysis was not feasible within the scope of this literature review. An overall assessment of methodological quality was undertaken for each included primary study using six key methodological criteria: setting description (I), description of the participants (II), sample size (III), definition of dependent and independent variables (IV), outcome data (V), and statistical analysis (VI). Each criterion was assessed dichotomously as positive (+) or negative (−). Based on these six parameters, the overall methodological quality score for each included study ranged from 0 to 6. Studies scoring below 3 were considered to be of low methodological quality. No primary study included in the final appraisal scored below this threshold; therefore, no study was excluded solely on the basis of the methodological quality score. Systematic reviews, meta-analyses, and clinical guidelines were included as supporting evidence but were not scored with this assessment, which was applied to primary studies. The methodological quality assessment and the main characteristics of the included primary studies are reported in Supplementary Tables S1, S2, respectively.

## Mandibular fractures

**Figure 1:**
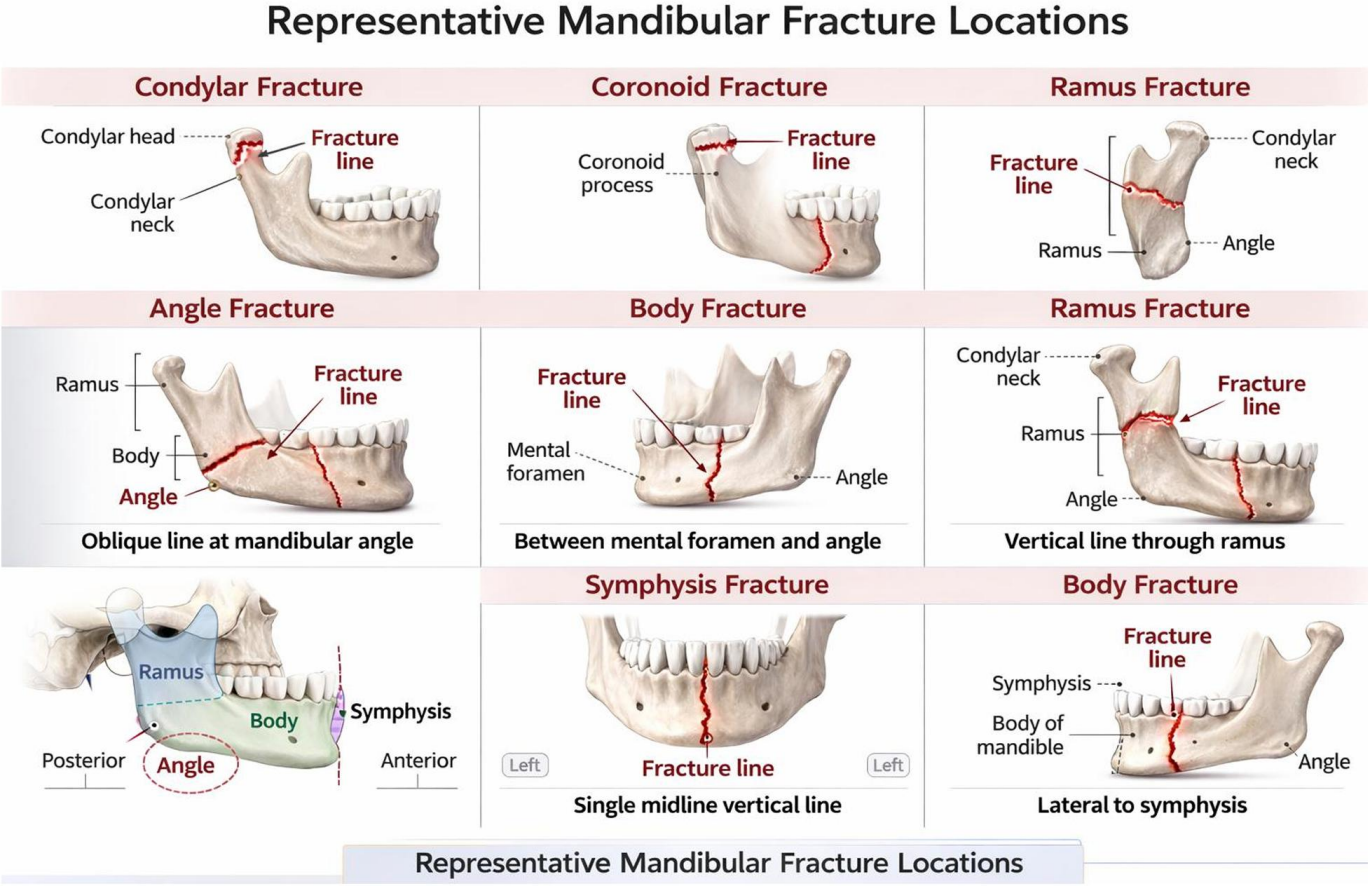
Schematic overview of mandibular fracture patterns.

For the purposes of this review, mandibular fractures are classified in accordance with the AO-CMF system as condylar and non-condylar fractures (13) (Figure 1).

### Condylar fractures

**Figure 2:**
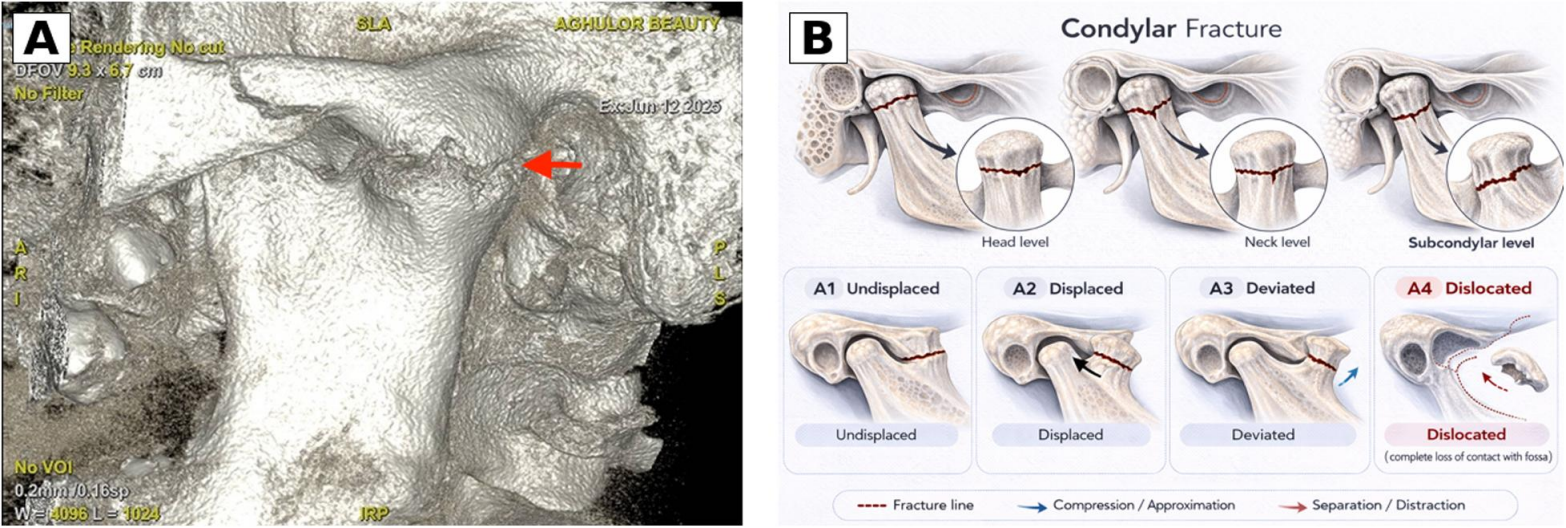
Condylar fractures. **(A)** Representative condylar fracture. **(B)** Schematic overview of condylar fracture patterns.

Condylar fractures (Figure 2A) are common, particularly in children and adolescents, and they have an incidence of approximately 45% of all mandibular fractures (18). The Strasbourg Osteosynthesis Research Group (SORG) classification divides condylar fractures into diacapitular (head), condylar neck, and condylar base fractures (19). This classification also assesses the degree of displacement of the fractured condylar head and thereby informs surgical or non-surgical treatment, commonly defining angulation of the condylar fragment relative to the mandibular ramus <10° or shortening of the mandibular ramus <2 mm as cutoff values (19). Moreover, according to Reddy et al., condylar fractures are defined as “displaced” when the condylar fragment has lost contact with the distal fracture fragment but the condylar head remains within the glenoid fossa; the term “dislocated” applies when the condylar head is no longer positioned within the glenoid fossa (16) (Figure 2B).

Clinically, patients with unilateral condylar fractures may present with facial asymmetry, malocclusion, deviation of the mandibular midline ipsilateral to the fractured side, reduced posterior facial height on the fractured side, and limitation of mandibular movements; these findings guide differential diagnosis and initial management (20–23). Bilateral condylar fractures are characterized by a reduction in posterior facial height on both sides, accompanied by retrognathia and labial incompetence. An anterior and lateral open bite may also be observed, with premature contacts in the posterior sectors and no deviation of the interincisal midline (Table 1) (24).

**Table 1:** Condylar fractures—clinical management.

Clinical question	Practical answer
Clinical examination	Occlusion, mandibular movements, deviation during mouth opening and closing, and pain; if ORIF is performed, assess wound status, nerve function, and fixation stability.
Imaging	Yes, OPT ± CT/CBCT; MRI only for persistent TMJ symptoms; periapical radiographs if an associated TDI is present.
Prophylaxis	Up to 24 h postoperatively in surgical cases; consider when dental trauma or extraoral communication is present.
Treatment	Conservative or surgical management according to displacement, function, and age.
Follow-up	Potential checkpoints, depending on fracture pattern and clinical course: within approximately 1 week, at 4–6 weeks, and at 3, 6, or 12 months when indicated; longer surveillance may be considered in growing patients. Assess TMJ function, ramus height, growth, and occlusion.

#### Imaging

Orthopantomography (OPT) is often used to initially assess the presence and location of the fracture, but additional imaging improves diagnostic accuracy in children and when condylar injuries are suspected (25). Cone-beam computed tomography (CBCT) provides better assessment of fracture displacement, ramus height, and associated fractures (3, 26). Magnetic resonance imaging (MRI) is reserved for patients with persistent temporomandibular joint (TMJ) symptoms or suspected disc/ligament injury (27). If condylar fractures occur in association with TDIs, periapical radiographs should also be performed (Table 1) (12)**.**

#### Prophylaxis

Condylar fractures may benefit from post-operative antibiotic prophylaxis continued for up to 24 h. Alternatively, antibiotic prophylaxis should be limited only to fractures involving a tooth socket or resulting from dental trauma (Table 1) (28).

#### Treatment

The choice between conservative and surgical management in condylar fractures remains debated, particularly in adults, and depends mainly on fracture level, degree of displacement, functional impairment, and patient-related factors (29–33). In adults, conservative treatment is primarily indicated for diacapitular fractures with condylar head deviation (<10°) or shortening of the mandibular ramus <2 mm and preserved occlusion (16, 19). Short-term intermaxillary fixation (IMF) may be considered in selected displaced or dislocated cases to allow for stabilization of the fracture fragments through bone callus formation, followed by early (2–3 weeks post-trauma) mobilization and physiotherapy to minimize the risk of ankylosis and functional limitation (16, 34, 35). In contrast, condylar neck and base fractures presenting with significant deviation, displacement, or dislocation—particularly when stable occlusion cannot be achieved—are generally managed with open reduction and internal fixation (ORIF) (16, 19, 36).

In growing patients (<16 years), functional orthopedic appliances represent the preferred approach for undisplaced condylar fractures and for selected fractures with deviation or dislocation of the condylar head, aiming to prevent growth disturbances and fracture-related malocclusions (20–23, 37–39). Orthopedic appliances promote condylar remodeling by applying distraction forces that displace the condyle away from the glenoid fossa contralateral to the injured side (40). By contrast, surgical management in pediatric and adolescent patients is usually limited to severely dislocated fractures that compromise occlusal stability (16, 36, 41). Systematic reviews and clinical series support conservative and functional treatment in growing patients, reporting favorable radiographic healing and functional outcomes (Table 1) (34, 42–45).

#### Follow-up

Because standardized evidence-based schedules are lacking, follow-up intervals should be individualized. Pragmatic reviews may be considered within 7 days, at 4–6 weeks, and at 3, 6, and 12 months (45–48). At each visit, static and dynamic occlusion (lateral excursions and protrusion), maximal mouth opening (MMO), mandibular symmetry or deviation, and TMJ symptoms may be assessed in both surgically and non-surgically managed patients (43). After ORIF, a review at approximately 1 week may include wound assessment for infection and evaluation of fixation stability (49). The 4–6-week visit may be used to assess early functional consolidation, whereas later reviews can evaluate residual malocclusion or TMJ dysfunction (46, 48). Radiographic healing in condylar fractures does not always correlate with functional recovery (43). In pediatric patients, follow-up beyond 12 months may be considered to detect growth disturbances or late mandibular asymmetry (Table 1) (20).

### Non-condylar mandibular fractures

Non-condylar fractures include injuries of the mandibular angle, ramus, body, symphysis, and coronoid process (13). Management depends on fracture displacement, occlusal stability, soft-tissue conditions, dentition status, and patient-specific factors, with recent literature summarizing outcomes for non-surgical and surgical strategies (50–53). For non-condylar fractures, radiographic appearance does not always predict functional outcome.

According to the AO-CMF classification, non-condylar mandibular fractures are categorized as “displaced” and “non-displaced” based on the presence or absence of spatial separation and loss of anatomical alignment between fracture segments (13). More specifically, “non-displaced” fractures exhibit a fracture line, but the bony fragments remain in anatomical alignment, with preserved cortical continuity and no significant gap, angulation, or translation (13). In contrast, “displaced” fractures show loss of anatomical alignment, which may include translation (lateral, medial, anterior, or posterior shift), angulation, rotation, vertical shortening (overriding fragments), and diastasis (gap formation) of the fracture fragments (13).

In addition, non-condylar mandibular fractures are defined as “favorable” and “unfavorable” based on the orientation of the fracture line and the vector of masticatory muscle forces. In favorable fractures, muscular forces promote fragment approximation and stability, whereas in unfavorable fractures, the fracture line orientation facilitates displacement of the segments under muscular pull (4, 13).

#### Mandibular angle fractures

**Figure 3:**
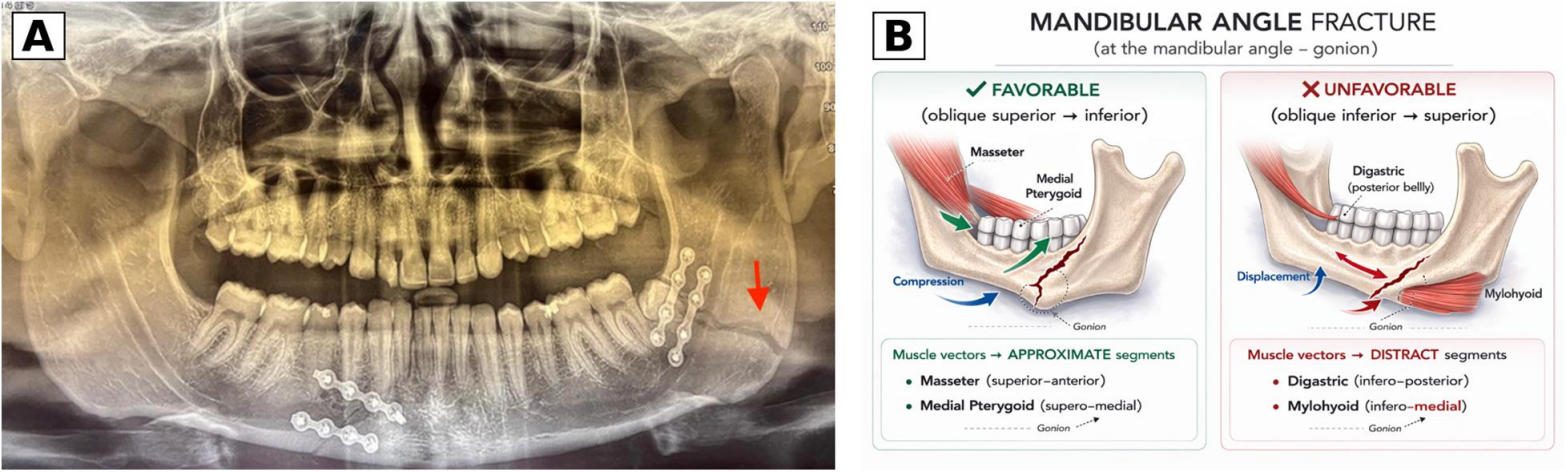
Mandibular angle fractures. **(A)** Representative mandibular angle fracture. **(B)** Schematic overview of mandibular angle fracture patterns.

The mandibular angle is one of the most frequently reported mandibular fracture sites (Figure 3), accounting for roughly 33% of mandibular fractures in several recent cohorts, although reported site distributions vary across populations and case series, and some reports identify the condyle as the most frequent site (54, 55). These injuries occur predominantly in young men and are strongly associated with interpersonal violence and alcohol misuse (51, 56–60). Unerupted or erupted third molars have been discussed as biomechanical contributors to angle/condyle fracture distribution, and biomechanical modeling supports the concept that third molars may affect local resistance (5, 61). Clinically, malocclusion, posterior open bite, and localized tenderness are common findings in mandibular angle fractures (Table 2) (1, 51).

**Table 2:** Mandibular angle fractures—clinical management.

Clinical question	Practical answer
Clinical examination	Malocclusion, pain, gingival lacerations, and assessment of the third molar region.
Imaging	Yes, OPT + CT/CBCT if displacement is uncertain; periapical radiographs if an associated TDI is present.
Prophylaxis	Up to 24 h postoperatively in surgical cases; consider when dental trauma or extraoral communication is present.
Treatment	Conservative management may be considered for non-displaced fractures; ORIF is generally favored when displacement or malocclusion is present.
Follow-up	Potential checkpoints: within 1–2 weeks and at 4–6 weeks; consider later review at 3–6 months for persistent symptoms or healing concerns. Assess occlusion, signs of infection, and neurosensory symptoms.

#### Mandibular ramus fractures

**Figure 4:**
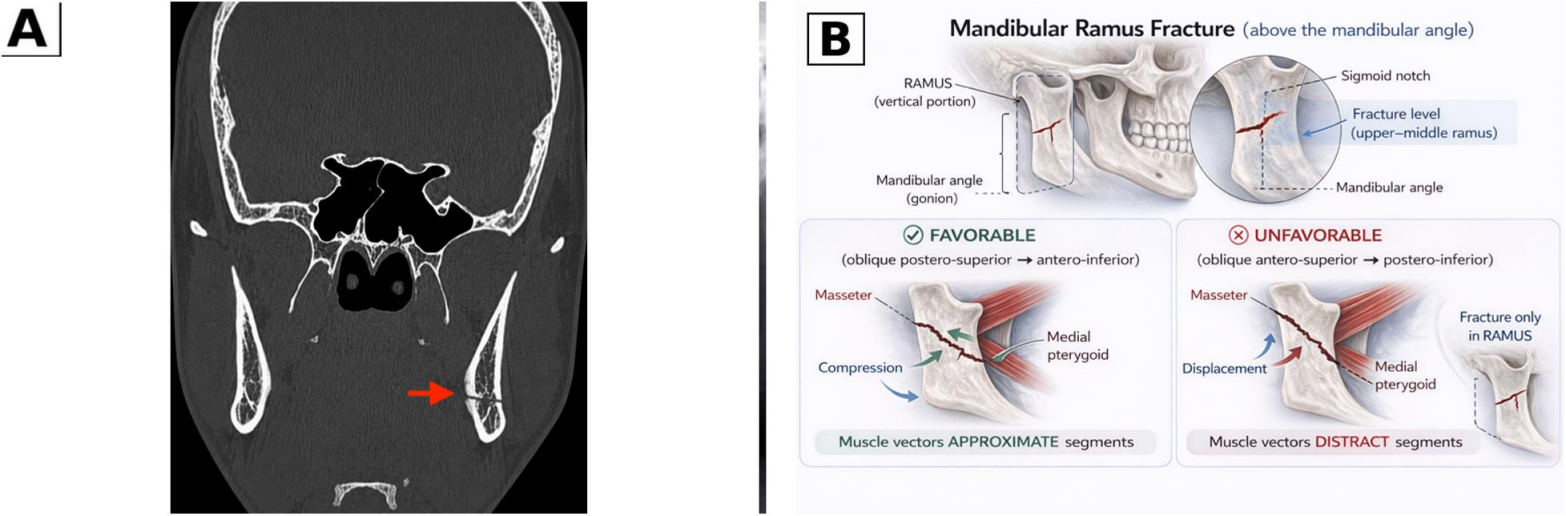
Mandibular ramus fractures. **(A)** Representative mandibular ramus fracture. **(B)** Schematic overview of mandibular ramus fracture patterns.

Ramus fractures (Figure 4) represent one of the least common mandibular fracture sites, second only to coronoid process injuries (62). They have an incidence of 3%–5% of all mandibular fractures (63) and may present with minimal displacement due to muscular stabilization, so clinical signs can be subtle (62).

Clinically, ramus fractures often preserve normal occlusion due to stabilization by the masseter, medial pterygoid, and the pterygomasseteric sling (56). Nonetheless, mandibular ramus fractures may exhibit mild displacement due to muscular forces (13). Trismus is common but may be mild because displacement is usually limited. Clinical examination typically reveals localized tenderness along the mandibular border, with possible step deformity or crepitus (56). Intraoral assessment may demonstrate gingival lacerations or molar mobility, particularly when a third molar lies within the fracture line (Table 3) (51).

**Table 3:** Mandibular ramus fractures—clinical management.

Clinical question	Practical answer
Clinical examination	Pain, limited function, and subtle malocclusion; periapical radiographs if an associated TDI is present.
Imaging	Yes, OPT; CT/CBCT if the diagnosis is uncertain.
Prophylaxis	Up to 24 h postoperatively in surgical cases; consider when dental trauma or extraoral communication is present.
Treatment	Often conservative when occlusion is stable and the fracture is non-displaced.
Follow-up	Potential checkpoints: within 1–2 weeks and at 4–6 weeks; consider later review at 3–6 months when clinically indicated. Assess occlusion, signs of infection, function, and neurosensory symptoms.

#### Mandibular body fractures

**Figure 5:**
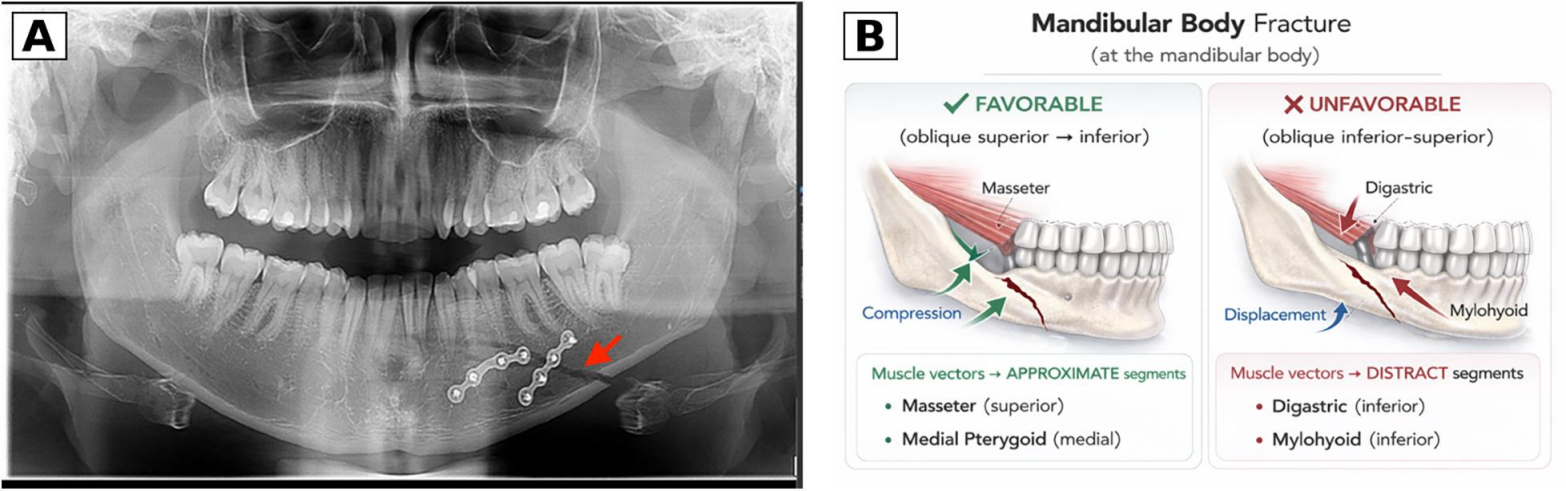
Mandibular body fractures. **(A)** Representative mandibular body fracture. **(B)** Schematic overview of mandibular body fracture patterns.

Mandibular body fractures (Figure 5) have an incidence of 12% to 22% of all mandibular fractures (5). They most commonly occur in young adults, with interpersonal violence, road-traffic accidents, and alcohol-related trauma being the primary etiological factors (58). Mandibular body fractures may involve the inferior alveolar nerve, and neurosensory deficits are common clinical concerns (1, 64). Clinically, posterior malocclusion and segmental mobility are frequent, with intraoral findings including gingival lacerations, vestibular hematoma, and dental or alveolar mobility (Table 4) (15).

**Table 4:** Mandibular body fractures—clinical management.

Clinical question	Practical answer
Clinical examination	Occlusion, segment mobility, and inferior alveolar nerve sensory status.
Imaging	Yes, OPT + CT/CBCT for displacement and canal assessment; periapical radiographs if an associated TDI is present.
Prophylaxis	Up to 24 h postoperatively in surgical cases; consider when dental trauma or extraoral communication is present.
Treatment	Conservative management if occlusion is stable and the fracture is non-displaced; ORIF if the fracture is displaced or associated with functional impairment.
Follow-up	Potential checkpoints: within 1–2 weeks and at 4–6 weeks; consider later review at 3–6 months according to healing and symptoms. Assess dentoalveolar integrity, neurosensory recovery, occlusal stability, and lower-lip and chin sensation.

#### Mandibular symphysis fractures

**Figure 6:**
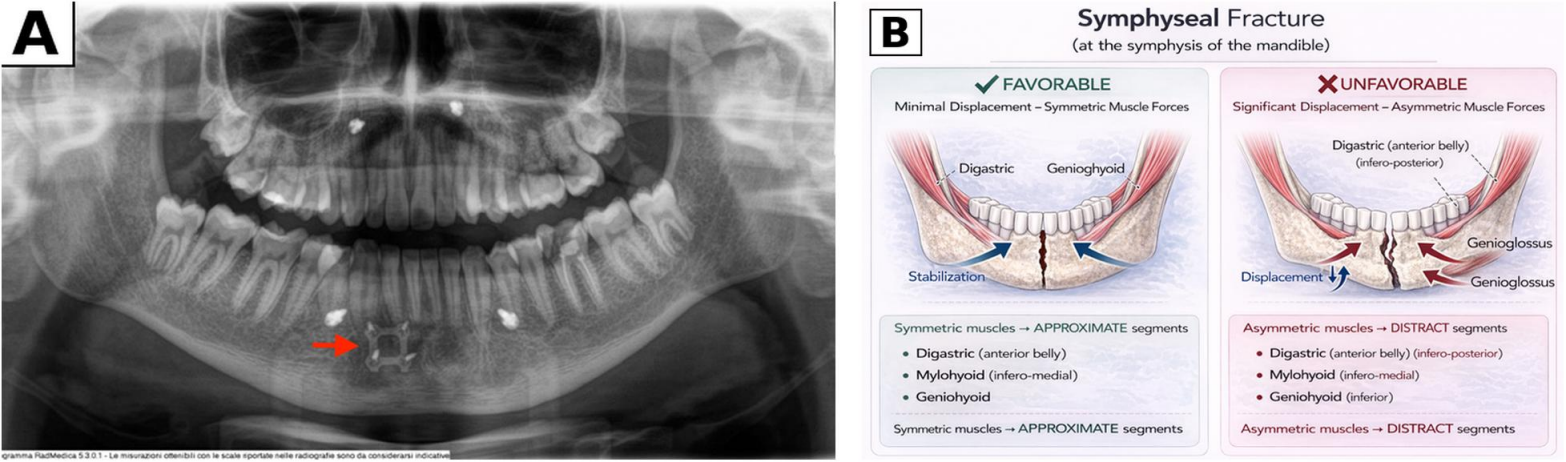
Mandibular symphysis fractures. **(A)** Representative mandibular symphysis fracture. **(B)** Schematic overview of mandibular symphysis fracture patterns.

Mandibular symphyseal and parasymphyseal fractures (Figure 6) have an incidence of 20%–30% of all mandibular fractures, frequently occurring in young adults and often associated with alcohol-related trauma (2). Clinically, symphyseal fractures often present with anterior malocclusion and instability, including mobility of the anterior dental segment and a midline diastema; treatment frequently requires fixation because of the mechanical demands of the anterior mandible (65). Close proximity to the mental foramen and frequent involvement of the inferior alveolar nerve contribute to neurosensory symptoms, particularly lower-lip paresthesia (Table 5) (64).

**Table 5:** Mandibular symphysis fractures—clinical management.

Clinical question	Practical answer
Clinical examination	Anterior malocclusion, segment mobility, and lower-lip paresthesia.
Imaging	Yes, OPT + CT/CBCT for displacement and comminution; periapical radiographs if an associated TDI is present.
Prophylaxis	Up to 24 h postoperatively in surgical cases; consider when dental trauma or extraoral communication is present.
Treatment	ORIF is often indicated when instability or malocclusion is present.
Follow-up	Potential checkpoints: within 1–2 weeks, at 4–6 weeks, and at 3–6 months; consider later review when clinically indicated. Assess occlusal stability, infection, and sensory symptoms.

#### Coronoid process fractures

**Figure 7:**
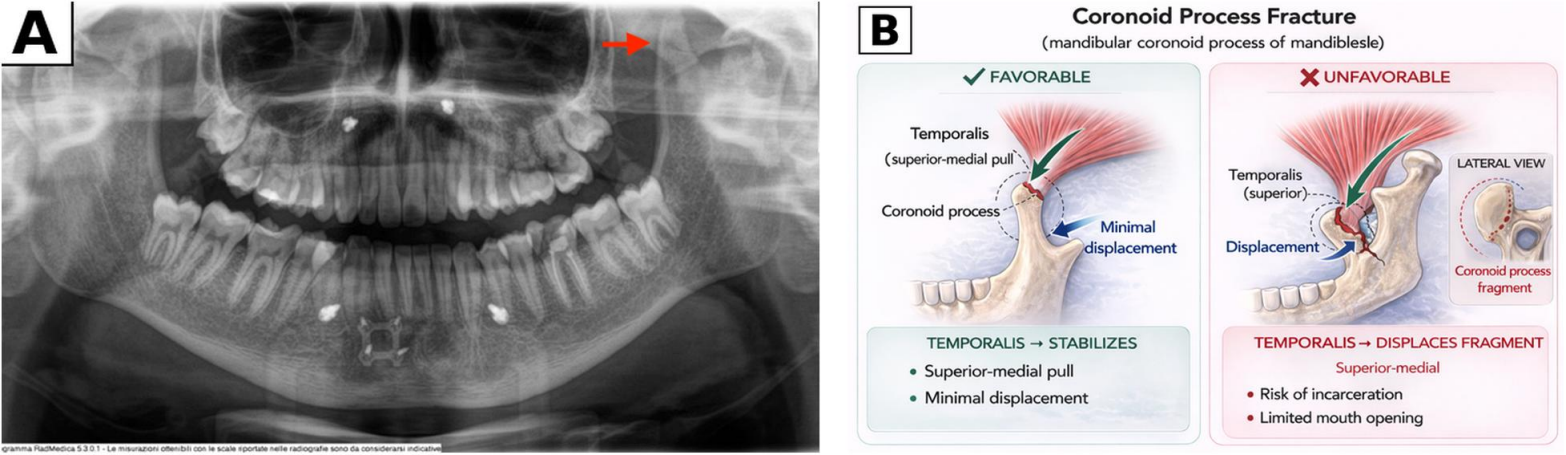
Coronoid process fractures. **(A)** Representative coronoid process fracture. **(B)** Schematic overview of coronoid process fracture patterns.

Coronoid process fractures (Figure 7) are uncommon injuries, representing 1%–4% of mandibular fractures in large epidemiologic cohorts (66). Their low incidence reflects the deep anatomical position of the coronoid process within the zygomatic arch and its protection by surrounding musculature (Figure 7) (67).

Coronoid fractures typically present with trismus rather than malocclusion. Restricted mouth opening, often with pain, deviation toward the affected side, and temporal tenderness, may indicate temporalis muscle involvement (Table 6) (67–69).

**Table 6:** Coronoid fractures—clinical management.

Clinical question	Practical answer
Clinical examination	Trismus, pain, and functional limitation.
Imaging	Yes; CT/CBCT is often the most informative modality.
Prophylaxis	Up to 24 h postoperatively in surgical cases.
Treatment	Conservative management in most isolated cases, with early physiotherapy.
Follow-up	Potential checkpoints: within 1–2 weeks, at approximately 6 weeks, and at 3–6 months; consider longer follow-up if trismus persists. Assess recovery of mouth opening and mandibular function.

#### Imaging

For non-condylar mandibular fractures, OPT is usually performed to locate the fracture, whereas three-dimensional imaging (CT/CBCT) is useful for assessing fracture displacement and comminution (3, 64, 70, 71). For mandibular body and symphysis fractures, CBCT also supports assessment of dentoalveolar injury, root integrity, fragment mobility, canal disruption, and the relationship of the fracture to the inferior alveolar canal (3, 64, 70, 71). If the fractures occur in association with TDIs, periapical radiographs should also be performed (Tables 2–6) (12).

#### Prophylaxis

Non-condylar mandibular fractures, if treated surgically, may benefit from post-operative antibiotic prophylaxis continued for up to 24 h after surgery. Fractures involving a tooth socket or resulting from dental trauma should be managed as open fractures and receive antibiotic prophylaxis as well (28). The duration of the prophylaxis depends on the type of contamination (Tables 2–6) (28).

#### Treatment

In non-condylar mandibular fractures with a non-displaced, favorable pattern and stable occlusion, conservative management with intermaxillary fixation (IMF), analgesia, a soft diet, and early physiotherapy may be appropriate. In contrast, displacement, malocclusion, comminution, instability, or functional impairment generally favor surgical management, usually with open reduction and internal fixation (ORIF) (51, 64). Fixation strategies usually include miniplates or locking plates, whereas 3D plates, reconstruction plates, or lag screws are used for high-load or comminuted fractures (51, 64). Cases of wire-free fixation have also been described (72, 73). In mandibular body and symphysis fractures involving the alveolar process and teeth adjacent to the fracture segments, stabilization of the bony fragments can be achieved by dental splinting techniques, whereby the involved teeth are secured to an arch bar or orthodontic wire (Tables 2–6) (74).

#### Follow-up

Because the available evidence does not define a uniform schedule, follow-up should be individualized. An early review within 1–2 weeks may be considered to assess occlusal stability, wound integrity, and early infectious complications (75). A 4–6-week evaluation may be used to assess primary consolidation, segment stability, and recovery of masticatory function (75). Longer follow-up at 3–6 months may be considered when clinically indicated to identify complications such as malunion or chronic infection (75). Moreover, in mandibular body and symphysis fractures involving the inferior alveolar nerve, dentoalveolar healing and neurosensory recovery should be monitored during follow-up (76, 77). Infection and other complications remain clinically relevant and are influenced by patient-specific factors, including smoking, systemic disease, and poor compliance (Tables 2–6) (1, 10, 64).

## Dental stabilization in traumatic dental injuries (TDIs) associated with mandibular fractures

In patients with mandibular fractures and associated traumatic dental injuries (TDIs), dental splinting should be planned in coordination with occlusal and surgical stabilization. The splint should be passive and flexible (e.g., orthodontic wire ≤0.016 inch bonded with composite resin), should not interfere with IMF/MMF devices, and should allow adequate oral hygiene and clinical monitoring (12). The objective is not merely mechanical stabilization of the tooth, but integration of the biological management of the TDI within the process of mandibular skeletal healing, avoiding both excessive immobilization and delays in the treatment of pulpal complications (12, 17).

The common biological rationale across different TDIs is to promote periodontal ligament and pulpal healing through passive and flexible immobilization of limited duration, avoiding unnecessary rigid or prolonged stabilization that may increase the risk of ankylosis or root resorption (12, 17). Stabilization of traumatized teeth should follow current dental-trauma guidance (12, 17, 78, 79), adapted to the maxillofacial surgical setting and the requirements of fracture stabilization (74, 80–82) (Table 7).

**Table 7:** Proposed, non-mandatory follow-up checkpoints in combined dento-mandibular trauma, adapted from IADT guidance; timing should be individualized according to injury type, treatment, symptoms, and clinical findings.

Potential checkpoint	Mandibular fracture	Specific TDIs	Clinical objective
Baseline (0–7 days)	Occlusal assessment, segmental stability evaluation, indication for conservative or surgical treatment; imaging according to clinical indication	Comprehensive clinical examination; baseline radiograph; photographic documentation; pulp sensibility testing (possible false-negative response during the first weeks)	Early identification of intrusion in mature teeth, avulsion, lateral or extrusive luxation in mature teeth, and cervical root fractures
7–10 days	Consider postoperative review after ORIF, including wound evaluation, occlusal stability assessment, signs of infection, and verification of MMF/IMF devices.	Clinical reassessment of traumatized teeth; no indication for endodontic treatment based solely on lack of sensibility response	Differentiate surgical-related symptoms from early signs of pulp necrosis
2 weeks	Clinical reassessment of occlusal stability; evaluation of intermaxillary immobilization if present	Consider splint removal for replanted teeth, extrusive or lateral luxation, and subluxation with significant mobility when clinical stability and injury-specific guidance support removal; perform clinical and radiographic reassessment.	Promote periodontal ligament healing while avoiding prolonged immobilization
3 weeks (in cases with IMF/MMF)	Consider removal of intermaxillary immobilization when indicated; verify stable occlusion and initiate mandibular mobilization as appropriate.	Reassess alveolar and cervical root fractures when splinting has been continued; consider extension to 4 weeks when clinically indicated.	Coordinate restoration of mandibular function and dentoalveolar stability
4 weeks	Evaluation of initial clinical consolidation; imaging only if symptoms or occlusal alteration are present	Consider splint removal for alveolar fractures and middle- or apical-third root fractures when clinically stable; perform clinical and radiographic reassessment of root fractures, complicated crown fractures, and luxation injuries.	Detect early pulp necrosis or inflammatory resorption
6–8 weeks	Evaluation of mandibular function, mouth opening, and possible neurosensory deficits	Clinical-radiographic follow-up in root fractures, complicated crown fractures, intrusions, and lateral or extrusive luxations in mature teeth	Identify initial necrosis of the coronal segment or external resorption
3 months (12 weeks)	Assessment of functional consolidation and occlusal stability	Consider clinical and radiographic review of intrusion, avulsion, and lateral or extrusive luxation; initiate endodontic treatment when clinical and radiographic evidence supports pulp necrosis or infection.	Detect late pulp necrosis and inflammatory resorption
6 months	Evaluation of functional and articular outcomes (including condylar region); neurosensory monitoring	Consider radiographic monitoring after avulsion, intrusion, severe lateral luxation, or root fracture according to injury-specific risk.	Identify replacement resorption or lesion progression
≥12 months	Assessment of definitive stability; in growing patients, monitoring of mandibular development	Consider clinical and radiographic review after avulsion, intrusion, lateral or extrusive luxation, root fracture, or complicated crown fracture according to risk and previous findings.	Confirm medium-term biological stability

## Integrated clinical algorithm for combined dento-mandibular trauma

**Figure 8:**
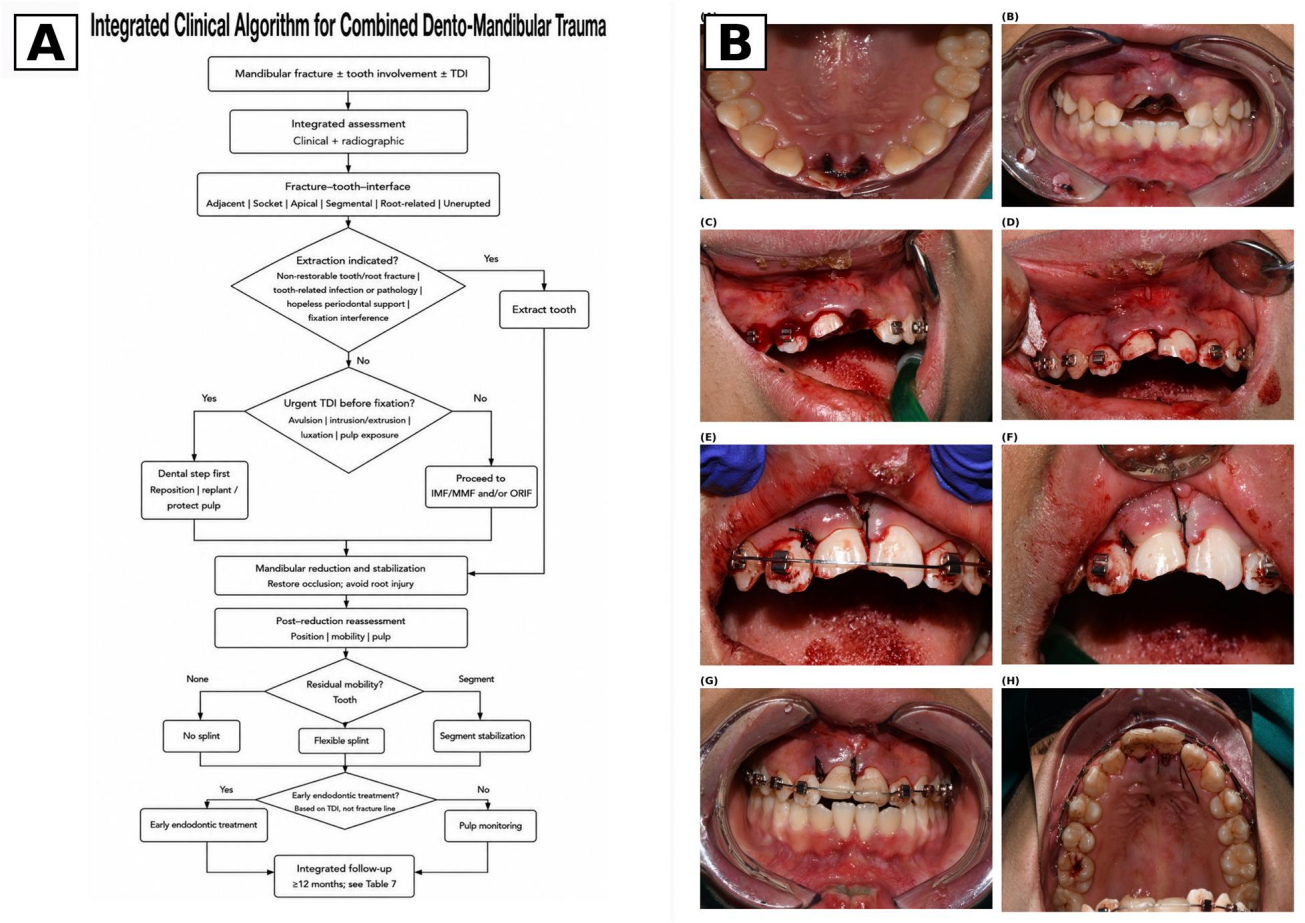
**(A)** Integrated decision-making algorithm for combined dento-mandibular trauma. The revised algorithm links systematic dental screening, combined fracture -dental assessment, tooth-in-fracture-line prognosis, time-sensitive traumatic dental injury management, skeletal/occlusal stabilization, endodontic timing, and long-term integrated follow-up. **(B)** Clinical examples andmanagement of traumatic dental injuries.

The revised pathway presented in Figure 8A uses the fracture–tooth interface as a central decision-support node linking systematic dental screening and combined fracture–dental assessment, fracture reduction, tooth retention, time-sensitive dental management, post-reduction mobility, splinting, and endodontic timing. Its distinctive elements are the anatomical characterization of the fracture–tooth relationship and reassessment after skeletal stabilization.

The algorithm represents an author-proposed synthesis of current maxillofacial, dental-trauma, and endodontic evidence. It is intended to support interdisciplinary clinical reasoning rather than to replace injury-specific guidelines, and its proposed anatomical patterns require prospective evaluation before they can be interpreted as validated prognostic categories (12, 17, 74, 78, 83–87).

Combined dento-mandibular trauma involves two distinct but interdependent stabilization targets. Mandibular stabilization aims to restore skeletal continuity, fracture-segment stability, and reproducible occlusion, whereas dental splinting is intended to maintain the position of a traumatized tooth or dentoalveolar segment while supporting periodontal ligament and pulpal healing (12, 17, 78, 83). These procedures therefore serve different biological and mechanical objectives. Dental splinting should not be regarded as a substitute for fracture fixation, while ORIF or IMF/MMF should be planned to avoid unnecessary compression of repositioned teeth, excessive immobilization, root injury, and loss of access for dental monitoring or treatment (74, 81).

The expression “tooth in the fracture line” refers to anatomically and biologically distinct conditions. For clinical decision support, the fracture–tooth relationship may be organized into six descriptive patterns: adjacent, when the fracture passes near the tooth without socket involvement; periodontal/socket, when the alveolar wall or periodontal ligament space is involved; apical, when the fracture reaches the root apex or adjacent neurovascular region; segmental, when the tooth-bearing alveolus moves with the mandibular fragment; root-related, when a root or crown–root fracture or displacement into the fracture gap is present; unerupted tooth, when the fracture involves the follicular region of an impacted or unerupted tooth (84, 86–89).

### Fracture–tooth interface and tooth retention

#### Adjacent interface

An adjacent interface does not require dental stabilization or endodontic treatment unless an independent traumatic dental injury is present. Management should therefore be based on the presence and type of any associated TDI rather than on the proximity of the fracture line alone (12, 85).

#### Socket interface

In a socket interface, the fracture line involves the alveolar wall and/or periodontal ligament space of an erupted tooth. Communication between the fracture and the oral cavity through the periodontal tissues identifies an open and potentially contaminated fracture (28, 74). However, socket involvement alone does not constitute an indication for extraction or prophylactic root canal treatment. The involved tooth should be assessed independently for displacement or luxation, root integrity, mobility relative to the fracture segment, periodontal and alveolar support, active infection, and possible interference with anatomical reduction, restoration of occlusion, plate positioning, or screw placement. An intact or restorable, periodontally maintainable, and non-infected tooth that does not interfere with fracture management may generally be retained and monitored (74, 80). Conversely, a root fracture or non-restorable crown–root fracture, severe tooth-specific mobility, extensive periodontal or alveolar-wall destruction, active odontogenic infection, or mechanical interference with reduction or fixation favor extraction (74, 86–90). Endodontic treatment should be determined by the associated dental injury, stage of root development, and longitudinal clinical and radiographic evidence of pulpal necrosis rather than by socket involvement alone (12, 85, 86).

#### Apical interface

An apical interface increases concern regarding damage to the pulpal neurovascular supply and therefore justifies closer clinical and radiographic surveillance. Nevertheless, a fracture line crossing the apical region should be considered a risk modifier rather than an automatic indication for endodontic treatment. Available observational evidence suggests that fracture displacement, associated tooth luxation, root integrity, infection, and postoperative stability may be more informative than the apical relationship considered in isolation (74, 86–90).

#### Segmental interface

In a segmental interface, movement of the tooth-bearing fragment must be distinguished from independent movement of the tooth within its socket. Apparent tooth mobility may therefore reflect instability of the mandibular or dentoalveolar segment rather than a true luxation injury. Reassessment after mandibular reduction and stabilization is required to determine whether residual mobility involves the individual tooth or the entire tooth-bearing segment and whether additional dental or segment-level stabilization is necessary (12, 74).

#### Root-related interface

A root-related interface requires injury-specific assessment. Potentially restorable horizontal root fractures may be retained, repositioned when necessary, and stabilized according to the level and mobility of the root fracture. Conversely, vertical root fractures, non-restorable crown–root destruction, active odontogenic infection, severe periodontal compromise, or interference with anatomical reduction, plate positioning, or screw placement favor extraction (12, 74, 85, 88, 89).

#### Unerupted tooth interface

In the available literature, this interface is represented primarily by impacted or unerupted third molars located within mandibular angle fracture lines. The presence of an unerupted tooth within the fracture line does not, by itself, mandate removal (60). Clinical and radiographic assessment should consider tooth displacement or dislocation, crown or root fracture, follicular or pericoronal pathology, active infection, mucosal communication, proximity to the inferior alveolar canal, and possible interference with anatomical reduction or osteosynthesis (60). A structurally intact, stable, and asymptomatic unerupted tooth that does not obstruct fracture treatment may generally be retained and monitored (60). Extraction should be considered when the tooth is fractured or displaced, is associated with active pericoronal or follicular disease or infection, communicates with the oral cavity, or prevents adequate reduction, plate placement, or fixation (74, 88). Because the available evidence does not support routine removal in every case, the decision should be individualized according to tooth condition, fracture anatomy, and surgical requirements (91).

If none of the above circumstances is present, retention is generally preferred when the tooth is restorable, periodontally maintainable, free of active infection, useful for occlusal guidance, and does not interfere with fracture reduction or fixation. Retained teeth require structured follow-up because pulp necrosis, inflammatory or replacement resorption, ankylosis, periodontal breakdown, marginal bone loss, increased mobility, and delayed extraction may occur. Because the evidence does not define a single mandatory duration, surveillance should be individualized; follow-up for at least 12 months may be considered, with extension according to the TDI type, root maturity, fracture pattern, and emerging clinical or radiographic findings (12, 17, 78, 83, 86–88).

### Dental splinting and coordination with mandibular stabilization

Time-critical TDIs (Figure 8B)—avulsion, intrusion, extrusion, lateral luxation, and displaced dentoalveolar fractures—should be managed as early as patient safety and fracture anatomy permit. The treatment sequence should be individualized. Dental repositioning or replantation may precede intermaxillary fixation (IMF) when an accurate and stable position can be achieved without compromising patient safety or fracture reduction (12, 17, 78, 83).

When no time-critical TDI requiring stabilization is present, intermaxillary fixation (IMF) and/or open reduction and internal fixation (ORIF) should generally precede the indicated dental management, which can be undertaken once fracture stabilization and occlusal control have been achieved (12, 85).

During ORIF or IMF/MMF, plates, screws, arch bars, and fixation wires should be positioned to avoid traumatized roots and preserve endodontic access, periapical imaging, oral hygiene, and clinical monitoring. Repositioned teeth should not be compressed or displaced by fixation devices, and severely mobile teeth or teeth of uncertain prognosis should not serve as the sole anchorage for fracture reduction. When interradicular fixation is planned and root proximity cannot be assessed reliably, appropriate radiographic verification should be obtained (74, 92–96).

After fracture reduction, the definitive stabilization plan should include evaluation of occlusion, fracture stability, tooth position, luxation or root fracture, and residual dentoalveolar mobility. In addition, independent tooth mobility should be distinguished from mobility of the entire tooth-bearing segment (12, 17, 83).

If no residual tooth or segment mobility remains, an additional dental splint may not be necessary. Conversely, minimal residual tooth mobility or mobility of the fracture fragment may require a flexible or more rigid splint, respectively. This approach avoids both undertreatment and unnecessary additional immobilization (12, 17, 74, 78, 81, 83).

### Endodontic timing and pulp monitoring

Endodontic management should be determined by the type of TDI, root maturity, pulp exposure, and serial clinical and radiographic findings rather than by the presence of a mandibular fracture alone. Early root canal treatment may be indicated or anticipated for replanted mature permanent teeth and for severely intruded mature teeth when recommended by current trauma guidance (12, 17, 78, 83, 85).

Extrusively and laterally luxated teeth should instead undergo serial pulp assessment unless pulp necrosis, infection, or infection-related resorption is diagnosed. In horizontal root fractures, endodontic treatment is not preventive and, when necrosis develops, is generally limited to the necrotic coronal segment. In immature permanent teeth, preservation of pulp vitality and the potential for revascularization and continued root development should be prioritized whenever biologically feasible (12, 17, 78, 83, 85).

A single negative pulp sensibility response during the early post-traumatic period does not establish pulp necrosis because temporary disruption of neural function may produce false-negative findings. Diagnosis should therefore be based on serial and concordant evidence, including symptoms, discoloration, swelling, sinus tract formation, tenderness, periapical changes, arrested root development, or infection-related external resorption. The same principle applies to teeth with an apical fracture–tooth interface: closer surveillance is justified, but root canal treatment should be initiated only when the specific injury or subsequent diagnostic findings support intervention (9, 12, 17, 85–87).

## Discussion and clinical perspective

Mandibular trauma care has evolved with advances in fixation systems and changing practice patterns, but substantial heterogeneity persists because fracture anatomy, dentition status, associated injuries, comorbidities, and clinical resources vary across settings (97, 98). Imaging choice, fixation strategy, and follow-up intensity should therefore be individualized rather than applied as uniform protocols (64, 70). In combined injuries, dental assessment is not ancillary, because TDIs may affect occlusal stabilization, fixation planning, tooth retention, and access for later dental treatment (6). However, evidence specifically addressing the fracture–tooth interface remains predominantly observational, and the prognostic value of individual anatomical relationships has not been prospectively validated (89).

International guidelines provide a strong framework for the management of specific fracture and dental-injury patterns, but direct comparative evidence for combined dento-mandibular trauma is limited. Recommendations concerning treatment sequence, splinting, endodontic timing, and coordinated follow-up are therefore partly extrapolated from separate bodies of maxillofacial and dental-trauma literature. The proposed algorithm should consequently be interpreted as a clinical synthesis intended to support, rather than replace, individualized judgment (1, 9, 12, 99).

Comparisons between conservative and surgical management are difficult to generalize because studies differ in fracture location, definitions of displacement, age groups, outcome measures, and follow-up duration (1, 50, 52, 76). These comparisons are also vulnerable to confounding by indication, because more displaced or unstable fractures are more likely to be selected for surgery. A similar selection effect applies to observational studies of teeth in the fracture line, since teeth selected for extraction are often infected, structurally compromised, or mechanically obstructive, whereas teeth selected for retention generally have a more favorable baseline prognosis (82, 86–91). Accordingly, the available comparisons should not be interpreted as demonstrating the universal superiority of one strategy. Numerical thresholds and follow-up intervals should be regarded as pragmatic decision aids rather than universally applicable rules, and the presence of an associated TDI may further modify the treatment pathway (6). The checkpoints proposed in Table 7 are therefore non-mandatory and should be adapted to injury type, root maturity, treatment modality, symptoms, access to care, and emerging clinical or radiographic findings.

A further unresolved issue is treatment sequencing when a time-critical TDI coexists with a fracture requiring urgent stabilization. No comparative trials establish whether dental repositioning or replantation should routinely precede or follow IMF/MMF or ORIF; the sequence must therefore be individualized according to patient stability, fracture anatomy, the time sensitivity of the dental injury, and the ability to maintain reproducible occlusion. Similarly, the follow-up checkpoints proposed in this review combine fracture-healing practice with injury-specific dental-trauma guidance rather than evidence from validated pathways for combined injuries. Their principal value is organizational, and a fixed schedule should not replace symptom-driven reassessment or local clinical judgment (1, 9, 12, 99).

## Limitations of the review

This review has limitations. First, the manuscript was designed as a literature review and clinical algorithm proposal, not as a systematic review or meta-analysis. Therefore, despite the addition of a structured search strategy, PICO framework, and eligibility criteria, the conclusions should be interpreted as clinically oriented guidance rather than formal graded recommendations.

Second, the available evidence on combined mandibular fractures and TDIs is heterogeneous, with much of the evidence derived from retrospective cohorts, case series, expert consensus, and guideline-based recommendations. Third, evidence specifically addressing the timing conflict between ORIF/IMF and endodontic treatment remains limited, and future prospective multicenter studies should evaluate tooth survival, pulp necrosis, resorption, ankylosis, fracture infection, and patient-reported outcomes using standardized core outcome sets.

## Conclusions

This literature review proposes an integrated clinical framework for combined dento-mandibular trauma. The revised algorithm emphasizes that mandibular fractures and TDIs should not be managed as isolated injuries: fracture stabilization, dental splinting, tooth retention/extraction, and endodontic timing must be coordinated from the initial assessment. Teeth in the fracture line should be evaluated individually rather than extracted routinely. Time-sensitive TDIs require early biologically appropriate management, while endodontic treatment should be guided by injury type, root development, and signs of pulp necrosis or resorption. Longitudinal follow-up should be individualized to detect both skeletal complications and late dental sequelae.
