# Supplementary material for: Integrated management of mandibular fractures and traumatic dental injuries: a literature review and clinical algorithm proposal
Source: Front Oral Health. 2026 Aug 3;7:1887405. doi: 10.3389/froh.2026.1887405 (PMC13478809; doi:10.3389/froh.2026.1887405)
Supplement: Supplementary file 1 [file Table1.docx]

**Supplementary Material**

Manuscript title: Integrated Management of Mandibular Fractures and Traumatic Dental Injuries: A Literature Review and Clinical Algorithm Proposal

**Supplementary Table 1. Methodological assessment of the included studies.**

An overall assessment of methodological quality was undertaken for each included primary study using six key methodological criteria: setting description (I), description of the participants (II), sample size (III), definition of dependent and independent variables (IV), outcome data (V), and statistical analysis (VI). Each criterion was assessed dichotomously as positive (+) or negative (−). Based on these six parameters, the overall methodological quality score for each included study ranged from 0 to 6. Studies scoring below 3 were considered to be of low methodological quality.

| **Study** | **Setting description** | **Description of the participants** | **Sample size** | **Variables** | **Outcome data** | **Statistical Analysis** | **DESCRIPTIVE SCORE (0-6)** |
| --- | --- | --- | --- | --- | --- | --- | --- |
| Eckelt et al. 2006 [46] | + | + | + | + | - | + | 5 |
| Danda et al. 2010 [48] | + | + | + | + | + | + | 6 |
| Hakim et al. 2018 [47] | + | + | + | + | + | + | 6 |
| Shiju et al. 2015 [32] | + | + | + | + | + | + | 6 |
| Merlet et al. 2018 [41] | + | + | + | + | + | + | 6 |
| Bansal et al. 2021 [45] | + | + | + | + | + | + | 6 |
| Oezmen et al. 1998 [27] | + | + | + | + | + | + | 6 |
| Throckmorton et al. 1999 [22] | + | + | + | + | + | + | 6 |
| Strobl et al. 1999 [20] | + | + | + | + | + | + | 6 |
| Rutges et al. 2007 [44] | + | + | + | + | - | + | 5 |
| Wang et al. 2019 [24] | + | + | + | + | + | + | 6 |
| Reddy et al. 2013 [16] | + | + | + | - | + | - | 4 |
| Arcuri et al. 2012 [49] | + | + | + | - | + | - | 4 |
| Xu et al. 2016 [38] | + | + | - | - | + | - | 3 |
| Boffano et al. 2012 [39] | + | + | + | - | + | - | 4 |
| Tuna et al. 2012 [21] | + | + | - | - | + | - | 3 |
| Tavares & Allgayer 2012 [23] | + | + | - | - | + | - | 3 |
| Staderini et al. 2025 [40] | + | + | - | - | + | - | 3 |
| Kuriakose et al. 1996 [72] | + | + | + | + | + | + | 6 |
| Xue et al. 2013 [75] | + | + | + | + | - | + | 5 |
| Gutta et al. 2014 [64] | + | + | + | + | - | + | 5 |
| Lamphier et al. 2003 [10] | + | + | + | + | + | + | 6 |
| Fahy et al. 2024 [59] | + | + | + | + | + | + | 6 |
| Arya et al. 2022 [76] | + | + | + | - | + | - | 4 |
| Pawar et al. 2022 [63] | + | + | - | - | + | - | 3 |
| Avelino et al. 2023 [88] | + | + | + | + | + | + | 6 |
| Walker et al. 2023 [84] | + | + | + | + | + | + | 6 |
| Deng et al. 2025 [60] | + | + | + | + | + | + | 6 |
| Patil 2012 [5] | + | + | + | + | + | + | 6 |
| Zhou et al. 2013 [6] | + | + | + | + | + | + | 6 |
| Kannari et al. 2022 [7] | + | + | + | + | + | + | 6 |
| Chacon et al. 2003 [25] | + | + | + | + | + | + | 6 |
| Kaeppler et al. 2013 [26] | + | + | + | + | + | + | 6 |
| Panicker et al. 2025 [71] | + | + | + | + | + | + | 6 |
| Saravanan et al. 2020 [2] | + | + | + | - | + | - | 4 |
| Kozakiewicz & Walczyk 2023 [18] | + | + | + | + | + | + | 6 |
| Anyanechi 2010 [54] | + | + | + | + | + | + | 6 |
| Bouguila et al. 2009 [55] | + | + | + | - | + | - | 4 |
| Rashid et al. 2013 [56] | + | + | + | - | + | - | 4 |
| Verma & Chambers 2015 [58] | + | + | + | - | + | - | 4 |
| Boole et al. 2001 [65] | + | + | + | - | + | - | 4 |
| Singleton et al. 2022 [98] | + | + | + | + | + | + | 6 |
| Sahoo et al. 2009 [89] | + | + | + | - | + | - | 4 |
| Widar et al. 2012 [93] | + | + | + | + | + | + | 6 |
| Kale et al. 2015 [67] | + | + | + | - | + | - | 4 |
| Genç et al. 2019 [69] | + | + | - | - | + | - | 3 |
| Carrillo Rivera et al. 2011 [66] | + | + | - | - | + | - | 3 |
| Alhumaid et al. 2024 [94] | + | + | - | - | + | - | 3 |
| Arcila et al. 2023 [90] | + | + | - | - | + | - | 3 |
| Hosgor et al. 2021 [86] | + | + | + | + | + | + | 6 |
| Sahoo et al. 2023 [89] | + | + | + | + | + | - | 5 |
| Gao et al. 2024 [87] | + | + | + | + | + | + | 6 |

**Supplementary Table 2. Summary of included studies.**

| **Authors** | **Year** | **Country** | **Type of study** | **Sample** | **Conclusions** |
| --- | --- | --- | --- | --- | --- |
| Eckelt et al. [46] | 2006 | Germany (multicentre) | Prospective randomized multicentre trial | 88 randomized; 66 evaluated (79 fractures) | ORIF produced better objective and subjective functional outcomes than closed treatment in displaced condylar fractures. |
| Danda et al. [48] | 2010 | India | Prospective randomized clinical trial | 32 patients (16 open; 16 closed) | No significant clinical differences were found between closed treatment and ORIF; however, ORIF achieved significantly better anatomical reduction. |
| Hakim et al. [47] | 2018 | India | Prospective randomized clinical trial | 30 patients (15 open; 15 closed) | ORIF improved anatomical reduction and mandibular function in moderately displaced fractures, although both groups achieved acceptable results. |
| Shiju et al. [32] | 2015 | India | Randomized controlled clinical trial | 50 patients (25 open; 25 closed) | ORIF yielded better anatomical reduction, occlusion, ramus-height maintenance, and less deviation than closed treatment. |
| Merlet et al. [41] | 2018 | France | Retrospective comparative study | 83 adults | Well-conducted functional treatment produced outcomes comparable to ORIF; surgery remained appropriate with substantial ramus-height loss. |
| Bansal et al. [45] | 2021 | India | Retrospective comparative study (paediatric) | 100 patients (77 closed; 23 ORIF) | Closed treatment was generally preferred in children, with broadly comparable outcomes to ORIF in selected cases. |
| Oezmen et al. [27] | 1998 | Germany | Comparative observational study | 30 patients (10 conservative; 20 surgical) | Both approaches achieved acceptable function; MRI demonstrated treatment-related differences in condylar and disc position. |
| Throckmorton et al. [22] | 1999 | USA | Prospective longitudinal comparative study | 22 patients plus matched controls | Masticatory patterns gradually adapted after bilateral condylar fractures, although abnormalities could persist despite clinically normal opening. |
| Strobl et al. [20] | 1999 | Austria | Prospective longitudinal cohort | 55 children | Conservative treatment generally produced favourable long-term function and remodelling, although radiographic asymmetry could persist. |
| Rutges et al. [44] | 2007 | Netherlands | Retrospective cohort with clinical re-examination | 60 records; 28 patients re-examined | Although subjective complaints were often limited, only 46% met the predefined success criteria, and residual clinical and occlusal abnormalities were common. |
| Wang et al. [24] | 2019 | USA | Retrospective cohort study | 38 patients with bilateral condylar injuries | Fracture pattern influenced prognosis; asymmetric condylar/subcondylar injuries showed the highest rate of adverse functional outcomes. |
| Reddy et al. [16] | 2013 | India | Retrospective descriptive study | 175 condylar fractures | Treatment should be individualized according to age, fracture level, displacement/dislocation, dentition, and occlusal stability. |
| Arcuri et al. [49] | 2012 | Italy | Retrospective case series | 14 patients | Endoscopically assisted repair was feasible in selected subcondylar fractures but was associated with procedure-specific complications. |
| Xu et al. [38] | 2016 | China | Case report | 1 paediatric patient | Conservative fixed-appliance orthodontic management restored occlusion, mandibular function, and facial symmetry after bilateral condylar fracture. |
| Boffano et al. [39] | 2012 | Italy | Prospective clinical case series | 14 children | Conservative treatment produced favourable clinical and functional recovery at one year in displaced unilateral condylar fractures. |
| Tuna et al. [21] | 2012 | Turkey | Case report | 1 growing patient | A conservative functional approach achieved satisfactory occlusal, functional, and radiographic healing at 2.5 years. |
| Tavares & Allgayer [23] | 2012 | Brazil | Case report | 1 paediatric patient | Orthodontic functional treatment corrected post-traumatic mandibular deviation and malocclusion after unilateral condylar fracture. |
| Staderini et al. [40] | 2025 | Italy | Case series | 2 cases | Both conservatively treated cases achieved favourable function and increased condylar bone density, although the radiological remodelling patterns differed. |
| Kuriakose et al. [72] | 1996 | United Kingdom | Retrospective comparative study | 266 mandibular fractures | Both fixation systems achieved fracture healing, but infection was significantly more frequent with miniplates (12.9%) than with rigid plates (2.3%). |
| Xue et al. [75] | 2013 | USA | Prospective randomized comparative trial | 18 enrolled; 13 completed analysis | The strut plate and Champy miniplate both achieved successful angle-fracture fixation; the strut was a safe alternative. |
| Gutta et al. [64] | 2014 | USA | Retrospective cohort study | 560 patients; 363 with complete data | Among 363 evaluable patients, the overall complication rate was 26.5%; hardware failure and infection were the most frequent complications, and 8.1% required revision. |
| Lamphier et al. [10] | 2003 | USA | Retrospective cohort study | 358 patients; 594 fractures | Complications occurred in 13.3% of fractures and varied according to fracture and treatment characteristics. |
| Fahy et al. [59] | 2024 | Ireland | Retrospective cohort study | 45 patients with isolated angle fractures | Complication (11.1%) and reoperation (1.8%) rates were low; smoking was significantly associated with postoperative complications. |
| Arya et al. [76] | 2022 | United Kingdom | Retrospective cohort study | 34 non-surgically managed patients | Minimally displaced non-condylar fractures were successfully managed non-surgically in 97% of selected patients with regular follow-up. |
| Pawar et al. [63] | 2022 | India | Case series | 4 patients | The four distinct ramus-fracture patterns required anatomy-specific ORIF strategies because fracture orientation and adjacent structures affected surgical access and fixation. |
| Avelino et al. [88] | 2023 | Brazil | Retrospective record review | 42 patients; 54 fractures | Teeth were retained in 64.8% of fractures; third molars were the teeth most frequently removed, supporting case-by-case rather than routine extraction. |
| Walker et al. [84] | 2023 | Switzerland | Retrospective cohort study | 184 patients; 189 teeth | Only 7.4% of retained fracture-line teeth developed tooth-related complications; intact teeth should generally be preserved unless a definitive extraction indication exists. |
| Deng et al. [60] | 2025 | China | Retrospective cohort study | 110 patients; 113 third molars | Stable third molars were retained, whereas severely displaced or fractured teeth were extracted; this trauma-specific strategy produced low postoperative complication rates. |
| Patil [5] | 2012 | India | Retrospective observational study | 190 fractures (110 condylar; 80 angle) | Incompletely erupted third molars increased angle-fracture risk and were associated with a lower frequency of condylar fractures. |
| Zhou et al. [6] | 2013 | China | Retrospective observational study | 294 patients with single fractures; 509 injured teeth | Dental injuries occurred in 43.5% of patients and were strongly related to the anatomical mandibular fracture site. |
| Kannari et al. [7] | 2022 | Finland | Retrospective cohort study | 118 paediatric patients | Dental injuries occurred in 34.7% of paediatric mandibular-fracture patients, were often multiple, and resulted in loss of at least one tooth in approximately 10%. |
| Chacon et al. [25] | 2003 | USA | Comparative diagnostic study | 37 children | Computed tomography was more accurate than panoramic radiography for diagnosing and characterizing paediatric condylar fractures. |
| Kaeppler et al. [26] | 2013 | Germany | Diagnostic accuracy study | 164 patients; 231 suspected sites | CBCT identified additional fractures or infractures at equivocal sites and changed treatment planning in 9.52% of cases. |
| Panicker et al. [71] | 2025 | Brunei | Retrospective imaging study | 200 maxillofacial fracture cases | Two-dimensional CT detected all fractures and outperformed 3D reconstructions diagnostically; 3D views aided visualization of displacement but missed 4% of fractures. |
| Saravanan et al. [2] | 2020 | India | Cross-sectional prevalence study | 50 patients | Road-traffic accidents accounted for 72% of cases, and the parasymphysis was the most frequent fracture site (26%). |
| Kozakiewicz & Walczyk [18] | 2023 | Poland | Retrospective observational study | 386 patients; 175 condylar fractures | Condylar-process fractures were identified in 175 of 386 patients (45.3%), with age-related differences in fracture level and pattern. |
| Anyanechi [54] | 2010 | Nigeria | Prospective observational study | 73 patients | Domestic-violence-related mandibular fractures predominantly affected young adults; patients in lower social classes were more likely to sustain multiple fractures. |
| Bouguila et al. [55] | 2009 | Tunisia | Retrospective 10-year review | 685 patients | Mandibular fractures mainly affected young men, with road-traffic accidents and interpersonal violence as leading causes. |
| Rashid et al. [56] | 2013 | United Kingdom | Retrospective epidemiological study | 1,261 patients; 1,994 fractures | Young men predominated; assault caused 72% of injuries, and the mandibular angle was the most frequent fracture site (30%). |
| Verma & Chambers [58] | 2015 | Australia | Retrospective epidemiological study | 111 patients; 159 fractures | Most patients were men aged 20-30 years; assault caused 61% of fractures, 60% were intoxicated, and the angle was the most common site. |
| Boole et al. [65] | 2001 | USA | Retrospective epidemiological study | 4,381 soldiers; 5,196 fractures | This large military cohort described the frequency and anatomical distribution of 5,196 mandibular fractures in 4,381 active-duty soldiers and related them to injury mechanisms. |
| Singleton et al. [98] | 2022 | Australia | Retrospective comparative epidemiological study | 393 patients/665 fractures; historical 205/376 | Over 30 years, IMF use decreased and semi-rigid fixation increased, with lower rates of malocclusion, non-union/delayed union, and permanent nerve injury. |
| Sahoo et al. [89] | 2009 | India | Retrospective cohort study (IMF screws) | 200 patients | IMF screws provided rapid and effective fixation in 200 patients, but root injury, screw loss, and mucosal overgrowth highlighted the need for careful placement and follow-up. |
| Widar et al. [93] | 2012 | Sweden | Retrospective comparative study | 123 patients (64 predrilled; 59 drill-free) | Dental-root injuries occurred only in the predrilled group; drill-free insertion significantly reduced the risk of permanent root damage. |
| Kale et al. [67] | 2015 | India | Retrospective case series | 11 patients | Linear fractures with minimal displacement were managed conservatively, whereas displaced fractures or those associated with other facial fractures were treated with ORIF. |
| Genc et al. [69] | 2019 | Turkey | Case report | 1 patient | Short-term IMF followed by physiotherapy produced stable healing and satisfactory anatomical, functional, and aesthetic recovery in a slightly displaced isolated coronoid fracture. |
| Carrillo Rivera et al. [66] | 2011 | Mexico | Case report | 1 patient | A non-displaced coronoid fracture associated with an orbitozygomatic fracture was managed conservatively because mandibular movement and occlusion were preserved. |
| Alhumaid et al. [94] | 2024 | Saudi Arabia | Case report (IMF root perforation) | 1 patient | Combined endodontic and surgical treatment successfully managed an IMF-screw-related root perforation and underscored the importance of careful screw positioning. |
| Arcila et al. [90] | 2023 | Colombia | Case report (tooth in fracture line) | 1 patient | A tooth in the fracture line was successfully retained, illustrating that preservation is feasible when the tooth is restorable and does not compromise reduction or fixation. |
| Hosgor et al. [86] | 2021 | Turkey | Retrospective clinical/radiographic cohort | 38 patients; 60 fracture-line teeth | Pulp survival was 69.8%; fracture displacement significantly influenced one-year tooth prognosis, and unnecessary endodontic treatment should be avoided. |
| Sahoo et al. [89] | 2023 | India | Retrospective cohort study | 56 patients; 66 fracture lines | Root fractures occurred in 32% of patients; all fractured multirooted teeth were extracted, while the four single-rooted teeth were retained after endodontic treatment. |
| Gao et al. [87] | 2024 | China | Retrospective cohort study | 78 patients; 83 teeth | Only 3 of 83 fracture-line teeth healed with complications, and most patients reported satisfactory outcomes, supporting selective retention. |

**Supplementary Table 3. Search strategy and eligibility framework.**

| **Component** | **Description** |
| --- | --- |
| Databases | PubMed/MEDLINE; Scopus; Web of Science Core Collection. Searches were complemented by manual reference-list screening and targeted searches of relevant guidelines/clinical resources. |
| Final search date | 10 June 2026. |
| Search period | Database searches: 1 January 2000 to 10 June 2026. Seminal pre-2000 studies identified through manual reference-list screening were retained when they provided clinically relevant information not adequately represented in more recent publications. |
| PubMed/MEDLINE search blocks | Three PubMed/MEDLINE search blocks were exported: (1) combined mandibular fracture and traumatic dental injury terms (n = 255); (2) teeth in the fracture line/tooth retention-extraction terms (n = 181); (3) ORIF, conservative/closed treatment, IMF/MMF, and outcomes terms (n = 904). |
| Scopus search blocks | Two Scopus search blocks were exported: (1) combined mandibular fracture and traumatic dental injury terms (n = 464); (2) teeth in the fracture line/tooth retention-extraction terms (n = 368). |
| Web of Science search blocks | Two Web of Science Core Collection search blocks were exported: (1) combined mandibular fracture and traumatic dental injury terms (n = 205); (2) teeth in the fracture line/tooth retention-extraction terms (n = 131). |
| Core fracture terms | “mandibular fracture”; “mandible fracture”; “mandibular trauma”; “maxillofacial trauma”; “condylar fracture”; “mandibular condyle fracture”; “mandibular angle fracture”; “mandibular body fracture”; “symphysis fracture”; “parasymphyseal fracture”; “ramus fracture”; “coronoid process fracture”. |
| Core dental trauma terms | “traumatic dental injury”; “traumatic dental injuries”; “dental trauma”; “dentoalveolar injury”; “dentoalveolar injuries”; “dentoalveolar fracture”; “tooth in fracture line”; “teeth in fracture line”; “teeth in the line of fracture”; “dental splinting”; “avulsion”; “luxation”; “intrusion”; “extrusion”; “pulp necrosis”; “root resorption”. |
| Core management terms | “open reduction and internal fixation”; “ORIF”; “closed treatment”; “closed reduction”; “conservative treatment”; “intermaxillary fixation”; “IMF”; “maxillomandibular fixation”; “MMF”; “tooth retention”; “dental extraction”. |
| Inclusion criteria | Clinical studies, systematic reviews, meta-analyses, epidemiological studies, case series with clinically relevant management data, and international guidelines/clinical resources addressing mandibular fractures, TDIs, teeth in the fracture line, fixation planning, splinting, endodontic timing, complications, or follow-up. |
| Exclusion criteria | Animal/in-vitro/biomechanical studies without direct clinical applicability; editorials/letters without management data; studies unrelated to mandibular fractures, TDIs, or combined dento-mandibular trauma; references that did not support the statement for which they were cited; duplicates or sources superseded by higher-level evidence. |

**Supplementary search strings**

**PubMed/MEDLINE main search:** (("mandibular fracture"[Title/Abstract] OR "mandible fracture"[Title/Abstract] OR "mandibular trauma"[Title/Abstract] OR "maxillofacial trauma"[Title/Abstract] OR "condylar fracture"[Title/Abstract] OR "mandibular condyle fracture"[Title/Abstract] OR "mandibular angle fracture"[Title/Abstract] OR "mandibular body fracture"[Title/Abstract] OR "symphysis fracture"[Title/Abstract] OR "parasymphysis fracture"[Title/Abstract] OR "ramus fracture"[Title/Abstract] OR "coronoid process fracture"[Title/Abstract]) AND ("traumatic dental injury"[Title/Abstract] OR "traumatic dental injuries"[Title/Abstract] OR "dental trauma"[Title/Abstract] OR "dentoalveolar injury"[Title/Abstract] OR "dentoalveolar injuries"[Title/Abstract] OR "dentoalveolar fracture"[Title/Abstract] OR "dentoalveolar fractures"[Title/Abstract] OR "tooth in fracture line"[Title/Abstract] OR "teeth in fracture line"[Title/Abstract] OR "teeth in the line of fracture"[Title/Abstract] OR "dental splinting"[Title/Abstract] OR avulsion[Title/Abstract] OR luxation[Title/Abstract] OR intrusion[Title/Abstract] OR extrusion[Title/Abstract] OR "pulp necrosis"[Title/Abstract] OR "root resorption"[Title/Abstract])) AND 2000:2026[pdat]

**PubMed/MEDLINE teeth-in-fracture-line search:** (("mandibular fracture"[Title/Abstract] OR "mandible fracture"[Title/Abstract] OR "mandibular angle fracture"[Title/Abstract] OR "mandibular body fracture"[Title/Abstract] OR "symphysis fracture"[Title/Abstract] OR "parasymphysis fracture"[Title/Abstract]) AND ("tooth in fracture line"[Title/Abstract] OR "teeth in fracture line"[Title/Abstract] OR "teeth in the line of fracture"[Title/Abstract] OR "tooth-bearing fracture"[Title/Abstract] OR "third molar"[Title/Abstract] OR "dental extraction"[Title/Abstract] OR "tooth retention"[Title/Abstract])) AND 2000:2026[pdat]

**PubMed/MEDLINE ORIF/conservative management search:** (("mandibular fracture"[Title/Abstract] OR "mandible fracture"[Title/Abstract] OR "mandibular fractures"[Title/Abstract] OR "mandible fractures"[Title/Abstract] OR "mandibular condyle fracture"[Title/Abstract] OR "mandibular condylar fracture"[Title/Abstract] OR "mandibular condyle fractures"[Title/Abstract] OR "mandibular condylar fractures"[Title/Abstract] OR "mandibular subcondylar fracture"[Title/Abstract] OR "mandibular subcondylar fractures"[Title/Abstract] OR "non-condylar mandibular fracture"[Title/Abstract] OR "noncondylar mandibular fracture"[Title/Abstract] OR "non-condylar mandibular fractures"[Title/Abstract] OR "noncondylar mandibular fractures"[Title/Abstract]) AND ("open reduction and internal fixation"[Title/Abstract] OR ORIF[Title/Abstract] OR "closed treatment"[Title/Abstract] OR "closed reduction"[Title/Abstract] OR "conservative treatment"[Title/Abstract] OR conservative[Title/Abstract] OR "intermaxillary fixation"[Title/Abstract] OR IMF[Title/Abstract] OR "maxillomandibular fixation"[Title/Abstract] OR MMF[Title/Abstract]) AND ("systematic review"[Publication Type] OR "meta-analysis"[Publication Type] OR randomized[Title/Abstract] OR randomised[Title/Abstract] OR cohort[Title/Abstract] OR prospective[Title/Abstract] OR retrospective[Title/Abstract] OR trial[Title/Abstract])) AND 2000:2026[pdat]

**Scopus main search:** TITLE-ABS-KEY (("mandibular fracture" OR "mandible fracture" OR "mandibular trauma" OR "maxillofacial trauma" OR "condylar fracture" OR "mandibular condyle fracture" OR "mandibular angle fracture" OR "mandibular body fracture" OR "symphysis fracture" OR "parasymphysis fracture" OR "ramus fracture" OR "coronoid process fracture") AND ("traumatic dental injury" OR "traumatic dental injuries" OR "dental trauma" OR "dentoalveolar injury" OR "dentoalveolar injuries" OR "dentoalveolar fracture" OR "dentoalveolar fractures" OR "tooth in fracture line" OR "teeth in fracture line" OR "teeth in the line of fracture" OR "dental splinting" OR avulsion OR luxation OR intrusion OR extrusion OR "pulp necrosis" OR "root resorption")) AND PUBYEAR > 1999 AND PUBYEAR < 2027

**Scopus teeth-in-fracture-line search:** TITLE-ABS-KEY (("mandibular fracture" OR "mandible fracture" OR "mandibular angle fracture" OR "mandibular body fracture" OR "symphysis fracture" OR "parasymphysis fracture") AND ("tooth in fracture line" OR "teeth in fracture line" OR "teeth in the line of fracture" OR "tooth-bearing fracture" OR "third molar" OR "dental extraction" OR "tooth retention")) AND PUBYEAR > 1999 AND PUBYEAR < 2027

**Web of Science main search:** TS=(("mandibular fracture" OR "mandible fracture" OR "mandibular trauma" OR "maxillofacial trauma" OR "condylar fracture" OR "mandibular condyle fracture" OR "mandibular angle fracture" OR "mandibular body fracture" OR "symphysis fracture" OR "parasymphysis fracture" OR "ramus fracture" OR "coronoid process fracture") AND ("traumatic dental injury" OR "traumatic dental injuries" OR "dental trauma" OR "dentoalveolar injury" OR "dentoalveolar injuries" OR "dentoalveolar fracture" OR "dentoalveolar fractures" OR "tooth in fracture line" OR "teeth in fracture line" OR "teeth in the line of fracture" OR "dental splinting" OR avulsion OR luxation OR intrusion OR extrusion OR "pulp necrosis" OR "root resorption")) AND PY=(2000-2026)

**Web of Science teeth-in-fracture-line search:** TS=(("mandibular fracture" OR "mandible fracture" OR "mandibular angle fracture" OR "mandibular body fracture" OR "symphysis fracture" OR "parasymphysis fracture") AND ("tooth in fracture line" OR "teeth in fracture line" OR "teeth in the line of fracture" OR "tooth-bearing fracture" OR "third molar" OR "dental extraction" OR "tooth retention")) AND PY=(2000-2026)

Database-specific yield. The searches identified 2,508 records (PubMed/MEDLINE n = 1,340; Scopus n = 832; Web of Science Core Collection n = 336), plus 7 additional records identified through manual/reference-list screening and targeted guideline/clinical-resource searches; 2,083 records remained after duplicate removal.
